# Moderate and Severe Blood Pressure Elevation Associated with Stroke in the Mexican Hispanic Population

**DOI:** 10.4236/health.2017.96068

**Published:** 2017-06-30

**Authors:** Derek Senior, Michael F. Osborn, Katherene Tajnert, Ahmed Badr, Alok Kumar Dwivedi, Jun Zhang

**Affiliations:** Department of Biomedical Sciences, Texas Tech University Health Sciences Center, El Paso, USA

**Keywords:** Blood Pressure, Risk Factors, Ischemic Stroke, Mexican Hispanic, Epidemiology

## Abstract

**Background:**

Stroke is the fourth leading cause of death in US. Amongst other factors such as age, sex, race, genetics, obesity, diabetes etc., hypertension continues to be the leading contributing factor towards stroke. Studies regarding stroke in Hispanics are sparse and inconclusive.

**Objectives:**

The objective of the present study is to investigate the potential association between blood pressure elevation and risk of ischemic stroke among the Mexican Hispanic population.

**Methods:**

A retrospective data analysis was carried out for a planned case-control study with case-control ratios of 1:2. Mexican Hispanic cases were from the *ElPasoStroke* database with diagnosed hypertension that had sustained an ischemic stroke (n = 505) and Mexican Hispanics diagnosed with hypertension who were stroke-free as controls from the 2005–2010 NHANES databases (n = 1010). In this analysis, we included subjects who had data on systolic, diastolic or mean arterial blood pressures for cases (327) and controls (772). In cases, blood pressure was determined by the initial admission measurement, and in controls, the first measured blood pressure was used. The unadjusted and adjusted effects of continuous measurements of systolic, diastolic and mean arterial blood pressure on stroke were determined using logistic regression analyses. Subjects were further classified into groups based on prehypertension and hypertension ranges, as established by the Joint National Committee on Prevention, Detection, Evaluation, and Treatment of High Blood Pressure (JNC7). Unadjusted and adjusted logistic regression models were also used to determine the effect of categorized blood pressures.

**Results:**

Our data indicate that per unit increase in systolic, diastolic or mean arterial blood pressure elevates the odds of stroke among the Mexican Hispanic population. Adjusted analysis of categorized blood pressures showed that mild or moderate/severe high blood pressure significantly associated with odds of stroke. Maintaining and controlling blood pressure at more stringent and lower levels, specifically lowering mean arterial pressure may effectively reduce the odds of ischemic stroke among the Mexican Hispanic population.

**Conclusion:**

Elevation of blood pressure increases the odds of stroke among the Mexican Hispanic population. Our results provide new strategies to manage the stroke prevention and health disparity issues among the Mexican Hispanic population.

## 1. Introduction

Stroke has remained one of the most significant causes of morbidity and mortality in the United States despite advances in treatment and management. As of 2011, it is the fourth leading cause of death in the United States [1]. The most noteworthy non-modifiable risk factors for stroke are age, sex, race and genetic factors. The risk of stroke substantially increases with age, particularly in those older than 65 years, in which 75% of all strokes occur [2]. Several modifiable risk factors that have been identified are hypertension, obesity, diabetes, metabolic syndrome, and hyperlipidemia. Among all of the modifiable risk factors the most prevalent is hypertension [3] [4] [5] [6]; million American adults (31%) have high blood pressure [7]. Billions of dollars each year are devoted to the total cost of care for patients affected by stroke. Stroke costs the U.S. an estimated $38.6 billion each year [8]. This total includes the cost of health care services, medications, and missed days of work.

In regard to race, stroke continues to have disproportionate morbidity and mortality rates in the minority populations [9] [10] [11] [12] [13]. Several factors have been shown to contribute to this aspect of health disparities [14]. For instance, minorities tend to have high rates of hypertension, diabetes mellitus and obesity, lower income, lower educational background, and report inadequate physical activity compared to non-Hispanic whites [11]. In addition, Hispanics have been found to be affected at younger ages and have the greater rate of recurrence of stroke [12]. The improvement on racial and ethnic health disparities in stroke awareness is urgently needed [13] [15] [16] [17] [18] [19].

The studies and data regarding stroke in the Hispanic population are generally sparse and often inconclusive. Overall, Mexican Americans make up the largest subgroup of Hispanic Americans but are frequently underrepresented in studies. Studies tend to group together all Hispanics [20] and do not take into account the various subgroups from Mexico, South America, and the Caribbean basin, individually. It has been established that those of Mexican origin must be considered independently from other Hispanic populations based on genetic differences [21] [22]. According to 2010 U.S. census data, 82% of the population of El Paso, Texas is Hispanic and predominantly of Mexican origin. Therefore, we consider this population unique and aim to study risk factor modifications, such as ideal goals for hypertension specific to this population. Even though there continues to be growing evidence that stroke risk factors differ between race groups, national guidelines for prevention of stroke focuses on risk factors in the general population do not address the specific differences of race groups. In our previous study [23], we evaluated the effect of metabolic syndrome on ischemic stroke in a case control study. Recently, some progresses in risk factors associated with ischemic stroke among Mexican Americans have been made [24] [25]. This study aimed to evaluate the association of blood pressures along with individual components of metabolic syndrome with ischemic stroke. Our specific aim was to evaluate the effect of blood pressure on ischemic stroke as compared with controls in Mexican Hispanic population only.

## 2. Objectives

The objective of the present study is to investigate the potential association between blood pressure elevation and risk of ischemic stroke among the Mexican Hispanic Population. Based on our previous finding that hypertension increases the odds of ischemic stroke nearly forty times in this population, we hypothesize that subtle elevation of ether systolic, diastolic or mean arterial blood pressure is associated with ischemic stroke among the Mexican Hispanic Population.

## 3. Patients and Methods

### 3.1. Study Design

A case-control study was conducted using Mexican Hispanic cases from the *ElPasoStroke* database with diagnosed hypertension that had sustained an ischemic stroke (n = 505) and Mexican Hispanics diagnosed with hypertension without a prior history of stroke as controls from the 2005–2010 National Health and Nutrition Examination Survey (NHANES) databases (n = 1010) consists of The inclusion criteria for this study is that the ischemic stroke subjects were identified with a diagnosis of cerebral thrombosis with/without infarction, cerebral embolism with/without infarction, and cerebral artery occlusion, unspecified with/without infarction, by searching through the *ElPasoStroke* database as we described before [23]. Data were collected from those admitted during 2005 to 2010 at University Medical Center, El Paso. Data from 2005–2010 NHANES of Mexican Hispanic subjects were used as the control group [26]. We retrospectively analyzed all the available cases and controls for the evaluating the relationship of blood pressures (BP) and ischemic stroke.

### 3.2. Study Variables

In cases, blood pressure was determined by the initial admission blood pressure measurement obtained in the *ElPasoStroke* data base. In controls, the first measured blood pressure during the study exam (out of three) was used in at tempt to match admission measurements of the cases. Subjects were further classified based on prehypertension and hypertension ranges, as established by the Joint National Committee on Prevention, Detection, Evaluation, and Treatment of High Blood Pressure (JNC7) (Table 1) [27], including, prehypertension and Stage 1 (mild) and Stage 2 (moderate) hypertension, and Hypertensive Crisis (Severe) based on the values of systolic blood pressure (SBP) and diastolic blood pressure (DBP). Other variables, such as gender, tobacco use, diabetes, heart disease, alcohol abuse, dyslipidemia, drug use and lung disease were also analyzed as cofactors with ischemic stroke.

### 3.3. Statistical Analysis

We examined the effect of continuous and categorized blood pressures on stroke (cases) as compared with controls. Continuous variables (e.g., age, blood pressure, arterial pressure, and body mass index) were described using mean and standard deviation (SD) while categorical variables with frequency and proportion. Unadjusted and adjusted logistic regressions were used determining the associations of blood pressures with cases as compared with controls. Multivariable logistic models were developed: 1) using continuous blood pressure measurements, 2) using mean arterial pressure, and 3) using JNC7 classifications. All the significant variables in univariate analysis at 15% level of significance were included in the stepwise multivariable logistic regression analysis. In the stepwise selection, the entry level section was specified at 10% while stay level selection was kept at 5%. Although we developed descriptive models, the goodness of fit of the developed logistic regression model was summarized using area under the curve (AUC), correct classification and Hosmer-Lemeshow (H-L) test to confirm interval validations. The high values of AUC and correct classification show high discriminatory ability of the developed model while non-significant p-value (p > 0.05) of H-L test shows improved fit. The results of logistic regression analysis were reported using odds ratio (OR) along with 95% confidence interval (CI) and p-values. P-values less than 5% were considered as significant results. All statistical analyses were carried out using SAS 9.3.

## 4. Results

We identified a total of 337 ischemic stroke subjects from whom an initial blood pressure was recorded at time of presentation to the hospital. The controls included a total of 772 Mexican Hispanic subjects with similar demographics. To identify the potential association with stroke, we measured the distribution of cases and controls and the level of systolic (SBP), diastolic (DBP) and mean arterial pressure (MAP) (Table 2).

Unadjusted association of continuous systolic, diastolic and mean arterial pressure measurements and categorized blood pressures along with other metabolic syndrome factors and baseline cofactors are also shown in Table 2. Per unit increase in systolic blood pressure increases the odds of ischemic stroke by 9% (OR: 1.09; 95%CI: 1.08 – 1.10, p < 0.0001). Odds of ischemic stroke was found to be 1.15 times more likely with per unit increase in diastolic blood pressure (OR: 1.15; 95%CI: 1.13 – 1.17, p < 0.0001). Odds of ischemic stroke was found to be 16% higher with per unit increase in mean atrial pressure (MAP) (OR: 1.16; 85%CI: 1.14 – 1.18, p < 0.0001). Categorized blood pressure according to JNC7 was also found to be associated with ischemic stroke. Both mild (OR: 18.31; 95%CI: 11.17 – 30.03, p < 0.0001) and moderate/severe (OR: 161.29; 95%CI: 95.32 – 272.93, p < 0.0001) categories had significantly higher odds of ischemic stroke compared to normal/prehypertension. In addition, the other considered cofactors were also associated with ischemic stroke except lung disease and alcohol abuse.

Table 3 shows the adjusted association of blood pressures with ischemic stroke. All the significant variables in univariate analysis were included in the stepwise multivariable logistic regression analysis. Since MAP was high collinear variable with systolic BP and diastolic BP, we excluded MAP from the first multivariable regression analysis. In adjusted analysis, we found systolic blood pressure and diastolic blood pressure were positively associated with ischemic stroke in model 1. In addition, the body mass index, diabetes, dyslipidemia, drug use, heart disease and tobacco use were also associated with ischemic stroke in model 1. The developed model 1 showed high discriminatory ability (AUC = 0.98, correct classification = 80%) and improved fit. Model 2 was developed to determine association of mean arterial pressure along with other cofactors with ischemic stroke compared to controls. All the factors were remained significant in model 2 as well. The predictive performance of the developed model 2 was found to be very similar as model 1. Model 3 also showed that JNC7 categorized blood pressure was found to be significantly associated with ischemic stroke. All the cofactors associated with ischemic stroke in model 1 & 2 were remained significant in model 3 as well along with JNC7 categorized blood pressures. The considered model indices demonstrated improved fit for model 3. In sum, categorized and quantitative forms of blood pressures significantly increased the odds of stroke compared to controls. Diabetes, and dyslipidemia also increased the odds of stoke compared to control. However, the higher level of BMI was negatively associated with odds of stroke. Super singly, age did not find to be associated with stroke in any models.

Table 4 displays the factors associated with ischemic stroke in each sub-category of blood pressure according JNC7 classification. Diabetes and heart disease were found to be associated with ischemic stroke in each blood pressure category. Body mass index was only associated with ischemic stroke in normal or mild categories of blood pressure. Dyslipidemia was only associated with stroke in moderate/severe category of blood pressure.

## 5. Discussion

Continuous blood pressure measurements have recently been used to predict the onset of ischemic stroke [28] and the severity of ischemic stroke [29] in other ethnic groups. Although Mexican Americans had a much higher cumulative incidence for ischemic stroke in the US [16], the relationship between continuous blood pressure measurements and ischemic stroke remain largely unknown to date. In this report, using univariate analysis, we demonstrate that all continuous blood pressure measurements (SBP, DBP and MAP) are significantly associated with increased odds of ischemic stroke, suggesting a strong association between elevated blood pressure and increased odds of ischemic stroke, which is further supported from our categorized blood pressure measurements (JNC7). These findings correlate well with our previous findings that demonstrated hypertension as the most significant independent risk factor for stroke in this population [23], which is also consistent with other studies in that borderline isolated hypertension is associated with increased odds of ischemic stroke [30].

Interestingly in our analysis, diastolic blood pressure appears to have a greater impact on ischemic stroke over systolic blood pressure. Further, joint systolic and diastolic blood pressure measured as mean arterial pressure (MAP) appears to be strongly associated with independent systolic and diastolic blood pressures.

In our adjusted analysis of categorized blood pressure measurements, even mild elevation in blood pressure significantly increases the odds of ischemic stroke, consistent with previous studies that there is increased odds of ischemic stroke with prehypertension, especially high range prehypertension [6], suggesting that in the Mexican Hispanic population, especially among those at high risk of stroke (obesity, diabetes, metabolic syndrome, and hyperlipidemia), blood pressure goals ought to be below mild blood pressure ranges. Extreme high blood pressures are well known to have a profound impact on stroke pathogenesis [31] [32] [33]. We found that remarkable 114-fold increased odds of ischemic stroke within moderate and severe hypertension group, even after adjustment of effects, there is still a significant increased odds of ischemic stroke within this group, indicating that blood pressure control goals ought to be focus on moderate and severe hypertension in Mexican Hispanic population.

This analysis was conducted only among Mexican Hispanics who had ever been diagnosed with hypertension, without knowledge of them receiving treatment or not. This could be a major factor in the results and it is probable to assume those who did not suffer stroke controlled their hypertension medically. Another issue was the use of a single blood pressure measurement, (at time of admission for stroke patients, at first check for controls) as blood pressure is elevated during ischemic injury as a protective measure to enhance perfusion [34]. Our sub-analysis showed that among moderate and severely large blood pressure group, dyslipidemia seem to be an important factor for stroke in this ethnic group. Diabetes and heart disease seem to be independent factors associated with ischemic stroke irrespective of the blood pressure categories. This further suggests that metabolic marker such as diabetes and dyslipidemia are critical for ischemic stroke especially in patients with moderate/severe blood pressures.

One of the major limitations of this study is a retrospective study. Retrospective nature of the study yielded some missing data on body mass index and missed to record some important risk factors such as smoking and cholesterol. Due to case-control design, this study could not estimate the prevalence of blood pressure sub-categories in Hispanic stroke patients. Further, the controls were not selected from the same clinic. Despite that, this study carries some significant strength such as this study extensively examined the blood pressure measurements along with other metabolic markers association with ischemic stroke in Mexican American population. In this population, majority of the ischemic stroke patients were observed with moderate/severe blood pressures followed by dyslipidemia and heart disease/diabetes. The effect size for mean atrial pressure was larger compared to systolic and diastolic blood pressure measures. Dyslipidemia was only associated with increase odds of ischemic stroke in moderate/severe blood pressure category.

If hypertension coexists with at least two other conditions (either obesity, elevated triglycerides, diminished high-density lipoprotein, dyslipidemia, or elevated fasting glucose), it becomes metabolic syndrome which is highly associated with stroke as a major risk factor [35] [36], particularly among patients with ischemic stroke [37] [38]. Actually, the metabolic syndrome has been defined as a major determinant of ischemic stroke in Asian population [39] [40] [41], we found the similar result among the Mexican Hispanic population [23].

It has been noted that hypertension is the most important modifiable risk factor for stroke [42]. However, managing DBP was the main focus to reduce stroke for a long time. Recently, SBP has become the target for stroke prevention [43]. Our data indicate that stroke prevention and blood pressure management should be focused on mild, moderate and severe hypertension groups, specifically lowering both systolic and diastolic pressures may effectively reduce ischemic stroke among the Mexican Hispanic population. For individuals with other risk factors for ischemic stroke such as diabetes, and heart disease, it is imperative to maintain blood pressure under mild ranges and possibly even lower. Specifically focusing on mean arterial blood pressure control may itself significantly reduce the odds of ischemic stroke ischemic stroke among the Mexican Hispanic population. Our results provide new strategies to manage the stroke prevention and health disparity issues among the Mexican Hispanic population.

## Figures and Tables

**Table 1.** 7^th^ Joint national committee hypertension classifications (JNC7).

Classification (JNC7)	Systolic BP (mmHg)	Diastolic BP (mmHg)
**Normal**	90 – 119	60 – 79
**Pre-hypertension**	120 – 139	80 – 89
**Stage 1 hypertension (mild)**	140 – 159	90 – 99
**Stage 2 hypertension (moderate)**	≥160	≥100
**Hypertensive Crisis (severe)**	>180	>110

**Table 2.** Summary and comparison of cofactors according to ischemic stroke and controls.

Variables	Control (N = 772) N (%)	Stroke (N = 337) N (%)	OR (95% CI)	P - value
**Age (years): mean (SD)**	43.39 (16.84)	61.78 (10.70)	1.08 (1.07, 1.10)	<0.0001
**BMI (kg/m**^**2**^**): mean (SD)**	29.35 (6.20)	28.87 (5.63)	0.99 (0.96, 1.01)	0.2366
**SBP (mmHg): mean (SD)**	122.56 (18.24)	180.20 (33.19)	1.09 (1.08, 1.10)	<0.0001
**DBP (mmHg): mean (SD)**	69.59 (11.45)	94.96 (19.01)	1.15 (1.13, 1.17)	<0.0001
**MAP (mmHg): mean (SD)**	87.24 (11.59)	123.37 (21.94)	1.16 (1.14, 1.18)	<0.0001
**JNC7**				
** Normal/Prehypertension**	637 (82.51)	26(7.72)	1.00	
** Mild**	99(12.82)	74(21.96)	18.31(11.17 – 30.03)	<0.0001
** Moderate and severe**	36(4.66)	237(70.33)	161.29 (95.32 – 272.93)	<0.0001
**Gender**				
** Male**	393 (50.91)	162 (48.07)	1.00	
** Female**	379 (49.09)	175 (51.93)	1.12 (0.87, 1.45)	0.3852
**Tobacco**				
** No**	499 (64.64)	193 (57.27)	1.00	
** Yes**	271 (35.10)	144 (42.73)	1.37 (1.06, 1.79)	0.0173
**Diabetes**				
** No**	742 (96.11)	124 ( (36.80)	1.00	
** Yes**	30 (3.89)	144 (42.73)	15.28(11.07, 21.09)	<.0001
**Heart Disease**				
** No**	742 (96.11)	193 (57.27)	1.00	
** Yes**	30 (3.89)	144 (42.73)	18.45(12.08, 28.20)	<.0001
**Alcohol Abuse**				
** No**	592 (76.68)	257 (76.26)	1.00	
** Yes**	180 (23.32)	80 (23.74)	1.02 (0.76, 1.38)	0.8782
**Dyslipidemia**				
** No**	605 (78.37)	164 (48.66)	1.00	
** Yes**	167 (21.63)	172 (51.34)	3.82 (2.91, 5.03)	<.0001
**Drug Use**				
** No**	559 (72.41)	318 (94.36)	1.00	
** Yes**	213 (27.59)	19 (5.64)	0.16 (0.10, 0.26)	<.0001
**Lung Disease**				
** No**	745 (96.50)	325 (96.44)	1.00	
** Yes**	25 (3.50)	12 (3.56)	1.02 (0.51, 2.04)	0.9578

* SBP: systolic blood pressure; DBP: diastolic blood pressure; MAP: mean arterial pressure; BMI: body mass index; SD: standard deviation; OR: odds ratio; CI: confidence interval.

**Table 3.** Adjusted associations of blood pressures with stroke as compared with controls.

	Model 1	Model 2	Model 3
	OR(95%C), p-value	OR(95%C), p-value	OR(95%C), p-value
**SBP (mmHg)**	1.06(1.04, 1.07), <0.0001		
**DBP(mmHg)**	1.09(1.06, 1.12), <0.0001		
**MAP(mmHg)**		1.16(1.13, 1.19), <0.0001	
**JNC7**			
** Normal/Prehypertension**			
** Mild**			13.21(6.67, 26.19), <0.0001
** Moderate and severe**			114.39(56.53, 231.48), <0.0001
**BMI(kg/m**^**2**^)	0.94(0.89, 0.99), 0.0146	0.94(0.89, 0.99), 0.0131	0.95(0.9, 0.99), 0.0178
**Diabetes—Yes**	6.33(3.34, 12.01), <0.0001	6.71(3.56, 12.63), <0.0001	6.08(3.45, 10.72), <0.0001
**Dyslipidemia—Y es**	1.87(1.02, 3.44), 0.0441	1.86(1.01, 3.41), 0.0461	2.00(1.16, 3.46), 0.0125
**Drug Use—Yes**	0.14(0.05, 0.43), 0.0005	0.14(0.05, 0.4), 0.0003	0.28(0.11, 0.72), 0.0086
**Heart Disease—Yes**	12.54(5.87, 26.82), <0.0001	12.83(5.98, 27.54), <0.0001	9.96(5.06, 19.59), <0.0001
**Tobacco—Yes**	2.63(1.42, 4.9), 0.0022	2.59(1.4, 4.8), 0.0025	2.19(1.26, 3.78), 0.0052
**AUC**	0.98	0.98	0.97
**H-L test (p-value)**	0.8042	0.8017	0.1747
**Correct classification**	89.60%	89.50%	87.5%

Model 1: adjusted effects of continuous blood pressures on stroke; Model 2: adjusted effects of mean arterial pressure according to JNC7 on stroke; Model 3: adjusted effects of categorized blood pressures according to JNC7 on stroke; SBP: systolic blood pressure; DBP: diastolic blood pressure; MAP: mean arterial pressure; BMI: body mass index; OR: odds ratio; CI: confidence interval; AUC: area under the curve; H-L: hosmer-lemeshow test.

**Table 4.** Factors associated with ischemic stroke compared to controls in each JNC7 category of blood pressure.

JNC7 category	Cofactors	OR	95	%CI	p-value
**Normal/Prehypertension**					
	**BMI (kg/m**^**2**^)	0.89	0.8	1	0.0462
	**Diabetes—Yes**	104.51	19.35	564.56	<0.0001
	**Heart Disease—Yes**	32.46	7.11	148.17	<0.0001
**Mild**					
	**BMI (kg/m**^**2**^)	0.89	0.82	0.96	0.0039
	**Diabetes—Yes**	3.11	1.31	7.33	0.0098
	**Heart Disease—Yes**	7.17	2.6	19.77	0.0001
**Moderate and severe**					
	**Diabetes—Yes**	2.86	1.23	6.67	0.0149
	**Dyslipidemia—Yes**	4.77	1.79	12.7	0.0017
	**Heart Disease—Yes**	7.96	2.26	27.98	0.0012

BMI: body mass index; OR: odds ratio; CI: confidence interval.
